# Analytical validation of a novel comprehensive genomic profiling informed circulating tumor DNA monitoring assay for solid tumors

**DOI:** 10.1371/journal.pone.0302129

**Published:** 2024-05-16

**Authors:** Daniel R. Zollinger, Elizabeth Rivers, Alexander Fine, Yanmei Huang, Joseph Son, Akshita Kalyan, Wren Gray, Golshid Baharian, Carly Hammond, Rosalyn Ram, Lindsay Ringman, Dina Hafez, Daniel Savel, Vipul Patel, Marc Dantone, Cui Guo, Merrida Childress, Chang Xu, Dorhyun Johng, Brett Wallden, Prapti Pokharel, William Camara, Priti S. Hegde, Jason Hughes, Corey Carter, Nicole Davarpanah, Viraj Degaonkar, Pratyush Gupta, Sanjeev Mariathasan, Thomas Powles, Sean Ferree, Lucas Dennis, Amanda Young

**Affiliations:** 1 Foundation Medicine Inc, Cambridge, MA, United States of America; 2 Natera, Austin, TX, United States of America; 3 Roche/Genentech, South San Francisco, CA, United States of America; 4 Barts Cancer Institute, Barts Experimental Cancer Medicine Centre, Queen Mary University of London, Barts Health, London, United Kingdom; CNR, ITALY

## Abstract

Emerging technologies focused on the detection and quantification of circulating tumor DNA (ctDNA) in blood show extensive potential for managing patient treatment decisions, informing risk of recurrence, and predicting response to therapy. Currently available tissue-informed approaches are often limited by the need for additional sequencing of normal tissue or peripheral mononuclear cells to identify non-tumor-derived alterations while tissue-naïve approaches are often limited in sensitivity. Here we present the analytical validation for a novel ctDNA monitoring assay, FoundationOne®Tracker. The assay utilizes somatic alterations from comprehensive genomic profiling (CGP) of tumor tissue. A novel algorithm identifies monitorable alterations with a high probability of being somatic and computationally filters non-tumor-derived alterations such as germline or clonal hematopoiesis variants without the need for sequencing of additional samples. Monitorable alterations identified from tissue CGP are then quantified in blood using a multiplex polymerase chain reaction assay based on the validated Signatera^TM^ assay. The analytical specificity of the plasma workflow is shown to be 99.6% at the sample level. Analytical sensitivity is shown to be >97.3% at ≥5 mean tumor molecules per mL of plasma (MTM/mL) when tested with the most conservative configuration using only two monitorable alterations. The assay also demonstrates high analytical accuracy when compared to liquid biopsy-based CGP as well as high qualitative (measured 100% PPA) and quantitative precision (<11.2% coefficient of variation).

## Introduction

Circulating tumor DNA (ctDNA) in blood is an emerging prognostic and predictive biomarker for managing patient treatment decisions, informing risk of recurrence and response to therapy in early and late-stage disease [1–4]. Risk-assessment strategies in the adjuvant and perioperative setting are limited, leading to uncertainty around treatment planning. Failure to specifically identify those at high risk of recurrence can result in both over or under-treatment and ultimately impacts patient survival outcomes and quality of life [5,6]. CtDNA-based disease monitoring can serve as a sensitive method for molecular residual disease (MRD) and can accurately assess risk to support or defer perioperative treatment [7,8]. In the setting of advanced disease, standard radiologic criteria for response have limitations in accuracy, sensitivity, and timeliness among others [9–12]. Recent evidence suggests that changes in ctDNA levels are associated with treatment response, long term clinical outcomes, and can predict progression earlier than radiographic imaging [13,14]. Multiple diagnostic ctDNA monitoring assay approaches have been developed and include both sequencing of tissue for informing the selection of somatic variants for personalized multiplex NGS assays [15–17] and tissue-naive approaches [14,18,19]. The design of each of these approaches results in different performance characteristics that enable monitoring in meeting different clinical needs. Personalized whole exome sequencing (WES)-based assays enable the identification of a high number of tumor-specific variants, thus increasing the assay’s theoretical performance [20,21]. Tissue-informed approaches often utilize sequencing of matched normal tissues or peripheral blood mononuclear cells (PBMC) for removal of mutations in the germline and those associated with clonal hematopoiesis (CH), likely increasing cost and potentially turnaround time. Tumor-naive assays such as panel-based hybrid-capture assays of cell-free DNA (cfDNA) alone have limited sensitivity when measuring ctDNA and can also be impacted by CH variants without sequencing of matched PBMCs [20,22–24] thus limiting clinical utility in patients with low shed tumors or early-stage cancers. However, the benefit of tumor-naive assays is they make monitoring available to patients without a viable tissue sample.

We sought to develop a tumor tissue-informed, personalized monitoring assay that is derived from the validated and widely adopted comprehensive genomic profiling (CGP) assay FoundationOne®CDx [25,26] to identify monitorable alterations. Using genomic information derived from the FoundationOne®CDx assay to identify monitorable alterations allows patients who may only have historical tissue CGP to be eligible for monitoring, regardless of whether the treating physician was intending to utilize monitoring at the initiation of treatment. Furthermore, we developed an algorithm that can select monitorable alterations derived from tissue-only CGP, thus keeping the overall assay’s performance comparable to other tissue-informed monitoring assays without the need for sequencing of normal tissue or PBMCs.

Here we present the development and analytical validation of the FoundationOne®Tracker assay and the variant selection algorithm that identifies monitorable alterations from tissue CGP. We demonstrate that the assay is applicable to a majority of patients with a viable tissue sample by analyzing existing tissue data for the number of monitorable alterations per patient. We demonstrate this applicability across cancer types and multiple patient demographics. The analytical performance of the assay is assessed demonstrating that it has robust specificity, sensitivity, accuracy, and precision.

## Materials and methods

### Assay overview

**Fig 1:**
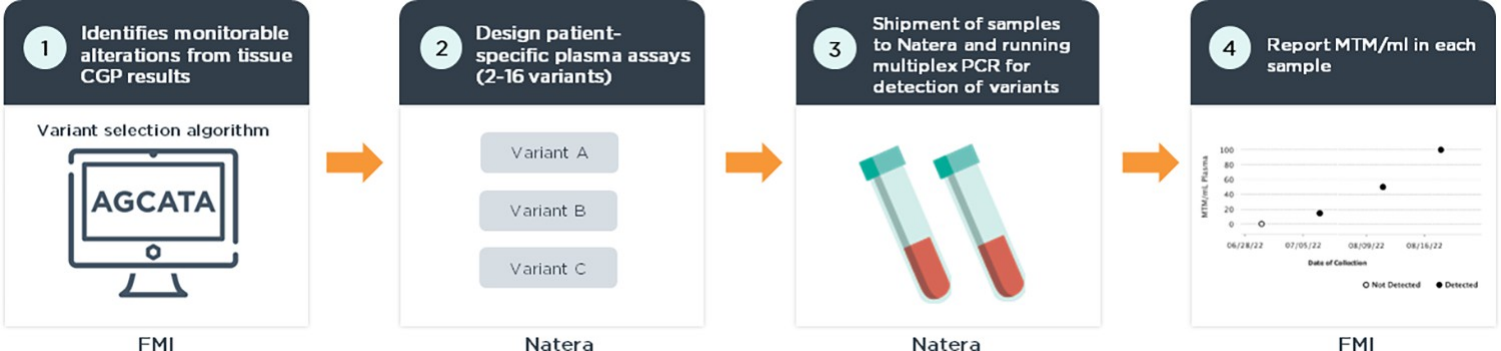
Assay overview. 1) The variant selection algorithm is used to identify monitorable alterations derived from tissue CGP. Tissue CGP and variant selection are performed by Foundation Medicine, Inc. (FMI). 2) Patient-specific primers are designed for up to 16 monitorable alterations per patient. Primer design is performed by Natera. 3) mPCR is performed on blood samples using the patient specific primer design in Natera’s CLIA/CAP approved laboratory. 4) mPCR data is analyzed to determine the MTM/mL for each sample. A report is generated by Natera and delivered by FMI.

Tissue and plasma samples from each patient are both analyzed by next-generation sequencing (NGS) assays following the workflow outlined in Fig 1. Tumor-specific variants are derived from tissue CGP using a proprietary variant selection algorithm. Alterations selected to have a high somatic probability are submitted to a plasma workflow based on the Signatera^TM^ assay [15,27]. cfDNA is extracted from patient plasma and personalized multiplexed PCR (mPCR) assays are used to quantify ctDNA at each timepoint. Quantification of ctDNA is reported as mean tumor molecules per mL (MTM/mL) [28]. The turnaround time for the FoundationOne®Tracker workflow is 7–10 days from sample receipt and variant design to providing the clinical report to the patient.

### Tissue CGP and variant selection algorithm

CGP of tumor DNA was performed retrospectively following methods described previously [25,26]. Briefly, DNA was extracted from formalin-fixed, paraffin-embedded (FFPE) tumor patient samples. CGP was performed as follows: DNA fragments were end-repaired, A-tailed, and adapters were ligated, followed by hybrid capture using a 324-gene panel, followed by NGS on the Illumina® HiSeq 4000. CGP was performed on patient samples in a Clinical Laboratory Improvement Amendments (CLIA)–certified, College of American Pathologists (CAP)–accredited, New York State–approved laboratory (Foundation Medicine Inc., Cambridge, MA). Approval for this study, including a waiver of informed consent and a HIPAA waiver of authorization, was obtained from the Western Institutional Review Board (Protocol No. 20152817). Data were accessed 1^st^ January, 2021.

The variant selection algorithm (Foundation Medicine, Inc.) is used to select short variants, including substitutions and short indels, from both exonic and intronic genomic regions in the data derived from CGP results for primer design while excluding non-tumor derived variants (germline, CH-derived, sequencing artifacts). This algorithm is based on a novel logistic regression model to predict probability of a variant being tumor-derived (somatic probability score) based on the difference between the observed variant allele frequency (VAF) and the expected germline VAF based on the copy number profile of the sample. Receiver operating characteristic (ROC) curve of the logistic regression model in classifying somatic variants identified in 116 matched tumor/normal pairs are shown in S1A Fig. An optimal somatic probability score threshold was chosen based on the ROC curve favoring minimization of false-positive rate (FPR). The algorithm then assesses variants based on the somatic probability score, allele frequency, and proprietary annotations. These annotations indicate whether a variant is a known driver, significant to tumor biology, or has unknown significance (VUS), and whether the alteration is listed in databases of known SNPs and CH variants (S1B Fig).

### CGP landscape analysis

Archival tissue CGP results from 53,371 patients were analyzed with the variant selection algorithm and the number of monitorable alterations was determined for each sample. These patients were not subject to primer design or the plasma workflow. Tumor mutational burden (TMB) was calculated as previously described [29]. Information for site of tumor origin, age, local or metastatic status, and cancer stage were provided by ordering physicians. The tumor type annotation for each case is derived from the test requisition form. In addition, a multistage pathology review was performed prior to and after sequencing. Prior to sequencing, board-certified pathologists on staff at Foundation Medicine, Inc. reviewed the submitted pathologic diagnosis of each case and examined hematoxylin and eosin (H&E)–stained slides. The H&E slide used for pathology review is the same as the FFPE tissue section dissected for sequencing. Tumor type assignment for each case was performed based on the submitting diagnosis and rereview of the H&E. Disease groupings were generated based on in-house disease tree mapping Disease ontology tumor types to Disease Groupings (S1 Table). Disease groups that contained fewer than fifty samples were mapped to an”other” category to improve data readability and interpretation. Ancestry was predicted using a SNP-based assignment [30]. All patient data was consented for research use.

### Plasma mPCR workflow

Samples with ≥2 monitorable alterations identified by the variant selection algorithm were submitted for primer design in the mPCR assay as previously described [15,31]. In brief, patient-specific variants were further filtered and ranked based on tumor VAF and expected plasma background error rates. Up to 16 variants were selected per patient to identify ctDNA in plasma samples. Patients with fewer than 2 alterations with successful primer design were not further assessed by the plasma workflow. cfDNA libraries were constructed followed by amplicon-based sequencing at an average NGS depth per amplicon of >100,000X on an Illumina platform. VAF was calculated for each of the targeted variants. MTM/mL was calculated using mean VAF, extracted cfDNA mass, and collected plasma volume as described previously [28]. Samples with ≥2 monitorable alterations detected were defined as ctDNA positive. mPCR and sequencing was performed on patient samples in a CLIA–certified, CAP–accredited, New York State–approved laboratory (Natera Inc, San Carlos, CA). Protocol approval was obtained from independent review boards or ethics committees at each site. Informed written consent was obtained from all patients. The patients/participants provided their written informed consent to participate in this study. No authors had access to information that could identify individual participants during or after data collection.

### Variant selection PPV

To determine the positive predictive value (PPV) of the variant selection algorithm selecting somatic alterations, matched FFPE tumor tissue and PBMCs were collected from 477 patients and analyzed with the tissue CGP assay defined above. Matched tissues were from 477 patients with advanced breast cancer, colon cancer, non-small cell lung cancer (NSCLC) (Indivumed Services), or muscle-invasive urothelial cancer (MIBC) [32]. Results from FFPE tumor tissue were processed with the variant selection algorithm and subject to the mPCR primer design as described above. Only targets with successful primer design were used for further analysis. Somatic alterations were defined as variants detected in tumor tissue and not detectable in the matched PBMC samples. Germline variants were defined as variants detected in FFPE tumors and in the matched PBMC samples with a VAF in the PBMC >30%. CH variants were defined as detected in FFPE tumors and in the matched PBMC samples with a VAF in the PBMC ≤30%. The limit of detection of the PBMC sequencing assay is expected to be similar to that described for the CGP tissue assay (1.8–11.8%) [25]. The 30% threshold for classifying CH vs germline was selected based on density distributions of allele frequencies of germline and CH variants constructed from a large amount of historical data from the CGP tissue assay; a 30% threshold minimizes the probability of mis-classification of germline as CH and vice versa. This threshold was used to estimate rates; however both CH and germline variants were subtracted for the analyses.

Protocol approval was obtained from independent review boards or ethics committees at each site. Informed written consent was obtained from all patients. The patients/participants provided their written informed consent to participate in this study. No authors had access to information that could identify individual participants during or after data collection. Data from MIBC samples was accessed on 14^th^ July, 2022. Data from advanced breast cancer, colon cancer, non-small cell lung cancer (NSCLC) were accessed on 11^th^ August, 2020.

### Analytical validation sample preparation

The assay was analytically validated with both cancer patient samples (NSCLC, breast and colorectal cancer, all were consented for use) and with contrived genomic DNA (gDNA) and cfDNA samples that were prepared using DNA from cancer cell lines. The majority of the samples used to statistically power the sensitivity, accuracy, and precision studies were contrived samples due to the limited availability of matched patient tissue and plasma samples with sufficient cfDNA mass and diversity of variants to support the planned analytical studies. Contrived gDNA and cfDNA sample pairs were prepared to serve as surrogates for solid tumor tissue gDNA and patient-matched plasma biopsy-derived cfDNA, respectively. A total of 4 unique contrived tumor samples were generated from different blends of 16 source cell line DNA samples (S2 Table). DNA from sets of 4 source cell lines were combined in equal mass ratio to obtain variants present at target VAF of 25% in each unique contrived tumor sample. Contrived tumor gDNA samples were processed with the variant selection algorithm to identify monitorable alterations.

From the 4 unique contrived gDNA samples, 4 undiluted contrived cfDNA samples were prepared by means of enzymatic fragmentation to a range of 100-250bp, from which 10-level contrived cfDNA dilution series (25%, 12.5%, 6.3%, 3.1%, 1.6%, 0.8%, 0.4%, 0.2%, 0.1%, 0.05%) were produced with two-fold serial dilutions with wild-type DNA (NA12878; Coriell Institute) as a diluent. Contrived gDNA and cfDNA paired samples were created from cancer cell lines that harbor a large number of variants. This allowed for multiple biological samples to be simulated from each contrived sample by designing multiple primer pools (of up to 16 primer sets in a pool). In addition to the contrived samples, patient samples were included for variant-level sensitivity, accuracy, and precision studies (Table 1). Patient samples were diluted with cfDNA from patients without a cancer diagnosis (termed unaffected) to match the VAF range of contrived samples. Studies were performed across the range of the cfDNA mass input specification of the assay (10ng to 66ng). Liquid CGP using a research use only version of FoundationOne®Liquid CDx [24] was performed for all undiluted samples. The analysis algorithm was modified to enable calling of variants from exonic and intronic regions as well as synonymous alterations. No authors had access to information that could identify individual participants during or after data collection. Data from patient samples were accessed between 30 August, 2021 and 13 October, 2022.

**Table 1:** Analytical validation studies.

Analytical Study		Assessment Level	Sample Type	Sample Size	Varied conditions
Specificity		Variant	Healthy donor plasma	1108 variants	
		Sample		520 samples	
Sensitivity		Variant	Contrived	1108 variants	VAF and mass input​
		Sample	Contrived	566 samples	Mean VAF and mass input
			Patient	16 samples	
Accuracy & Linearity		Variant	Contrived	5768 variants	VAF and mass input​
		Sample	Contrived	2864 samples	Mean VAF and mass input
Precision** **	Qualitative Repeatability	Variant	Contrived	186 sets of variants in triplicate	VAF range
			Patient	26 sets of variants in triplicate	
	Qualitative Reproducibility	Variant	Contrived	522 sets of variants in triplicate	VAF and operating conditions​
	Quantitative Reproducibility	Variant	Contrived	179 sets of variants in triplicate	VAF and operating conditions​
		Sample		66 sets of samples in triplicate	MTM/mL and operating conditions​

### Plasma workflow specificity

Plasma from 133 unaffected donors were analyzed by randomly selecting primers derived from 4,000 patient FFPE tumor samples previously evaluated with CGP from a variety of tumor types. Each plasma sample was used to test 4 different sets of 16 variants, the maximum number of variants per assay. A total of 520 unique plasma/primer sets (samples) that passed quality control metrics were used in the analysis. This approach was limited to testing the likelihood of false positives (FP) arising from technical sources (*e*.*g*. PCR error) in the plasma workflow as unaffected patients would not have matched FFPE tumor tissue. A study to determine the PPV of the variant selection algorithm is described above.

### Combined specificity estimate

The combined specificity of the variant selection algorithm and plasma workflow at the sample-level was calculated using the following equation:

Combined Specificity = 1—Pr(False Positives)

= 1—Pr{≥2 technical FP variants} * Pr{number of FP germline or CH variants = 0}
• Pr{≥1 technical FP variants} * Pr{number of FP germline or CH variants = 1}
• Pr{number of FP germline or CH variants = 2}

Lower (LB) or upper bounds (UB) of 90% Clopper-Pearson confidence intervals (CI) were used to estimate the lower bound of an estimate of combined specificity, representing the most conservative specificity of the assay. A point estimate (PE) of combined specificity was generated using the individual point estimates instead of bounds.

Combined Specificity

= 1 - (1- LB of the technical specificity estimate) * (UB of rate of 0 FP germline or CH)
• (UB of rate of ≥1 technical FPs) * (UB of the rate of 1 FP germline or CH)
• UB of the rate of 2 FP germline or CH variants

### Sensitivity

The sensitivity of the assay was evaluated at the variant and sample-level with contrived samples and patient samples across a range of intended VAFs (0.1, 0.2, 0.4, 0.8, 1.6, 3.1, 6.25%). Intended VAF levels were chosen to represent VAF levels targeted for homozygous variants. Upon VAF-targeted dilution, a range of VAF values was expected due to clonality, copy number variation, and zygosity. Expected VAF was calculated using dilution factors applied to the VAFs determined from liquid CGP of undiluted contrived cfDNA samples. Variant-level sensitivity was calculated for variants with expected VAF ≥0.3% and <0.5%. Sample-level sensitivity (ctDNA positive or negative status at a given MTM/mL range) was evaluated using data from the diluted contrived cfDNA samples described above using simulated samples in the range of 5–10 MTM/mL. Each simulated sample was created by randomly selecting 2 variants from a contrived cfDNA sample to evaluate the most challenging condition for sample-level detection (i.e. there is a lower probability of detecting 2 variants out of 2 than 2 out of 16 variants).

### Accuracy and linearity

In order to assess analytical accuracy (i.e. orthogonal concordance) and linearity, liquid CGP was also performed on all samples in this study. Accuracy was assessed using the slope and intercept results from a Passing-Bablok regression of FoundationOne®Tracker vs liquid CGP measured VAF for contrived sample variants falling within the liquid CGP 0.5–20% VAF range.

The variant-level linearity analysis examined the proportional relationship between FoundationOne®Tracker reported VAF and the respective intended VAF. Samples were generated from 3 contrived cfDNA mixtures (Mix A, B, and C, S2 Table) and were tested at 7 different intended VAF levels (0.4, 0.8, 1.6, 1.3, 6.25, 12.5, 25%), at 3 intended cfDNA input levels (10, 33, 66ng), and with 8 primer pools. Linear regression analysis was performed separately for each input level. In each case, weighted regressions were performed in order to correct for heteroskedasticity, or non-constant variance, over the range of analyzed VAF values. For each regression analysis, a power function model of the relationship between measured variance and intended VAF value was individually determined by obtaining coefficient estimates using least squares regression on log-transformed VAF variance estimates vs log-transformed intended VAF values. These estimates of the variance function were then used to obtain regression weights, determined as the inverse variance of VAF at the relevant intended VAF values in the weighted regression fits.

Variant and sample-level linearity at each DNA input level was examined by fitting three different linear models, regressing the VAF measurements on the first-order, second-order, and third-order nested polynomial functions of the intended VAF. The coefficient estimates were examined for each different model fit and individual t-tests were performed at the 5% significance level to test the null-hypothesis that each coefficient estimate is equal to zero.

### Precision

Conditions for testing the repeatability of qualitative variant detection were assessed with sets of 3 sample replicates (triplicates) processed by the same operator, using the same reagent lots, sequencing instruments, and on the same day. DNA input masses were 33 ng and 20 ng for contrived and patient samples, respectively. Only variants where the median VAF of the triplicate set was ≥0.5% (the lower limit of detection tested above) were included in the analysis as reliability of detection decreases below the limit of detection. The reproducibility of qualitative variant detection was assessed with sample replicates processed under 3 unique combinations of the operator, reagent lots, sequencing instrument, and day. Reproducibility of contrived samples was tested at the minimum (10 ng) and maximum (66 ng) input masses for the assay. Patient samples were tested at 20 ng input mass.

To assess quantitative precision, coefficient of variation (CV) was calculated from three separate runs with unique combination of the operator, reagent lots, sequencing instrument, and day. Each run had 6 replicates in each intended VAF bins instead of 3 to facilitate statistical analysis. Since there is no analytical formula to obtain uncertainty and CIs for the overall mean CV across the intended VAF range using the hierarchical procedure of averaging, a bootstrap-based approach was implemented.

## Results

### Variant selection algorithm

**Fig 2:**
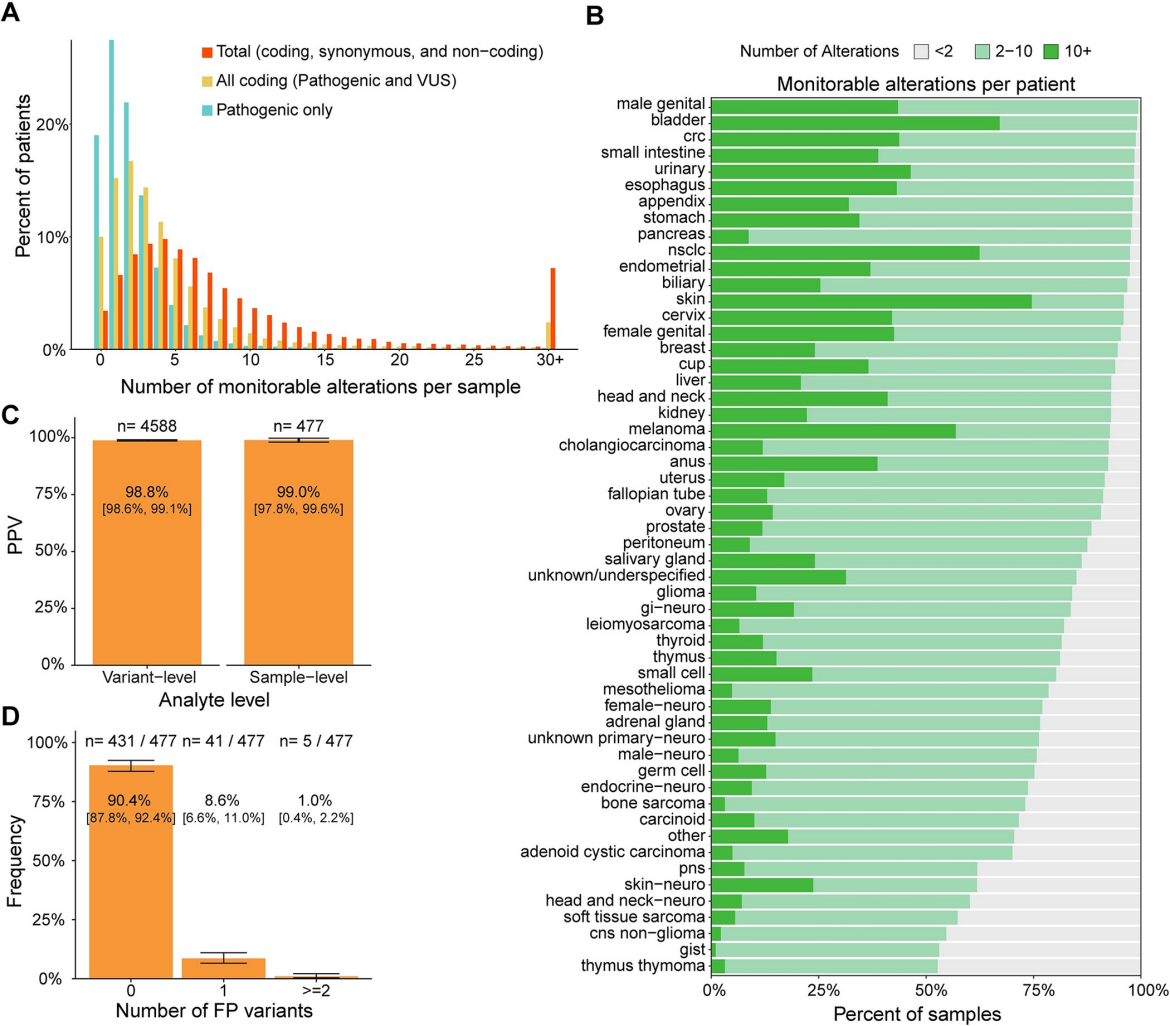
Variant selection algorithm. A) Percentage of patients by number of monitorable alterations and by variant status. B) Number of monitorable alterations designed per patient from historical tissue CGP across tumor types based on Foundation Medicine disease classification (See S1 Table for disease mapping). C) PPV of variant selection algorithm at the variant and sample-level. D) Frequency of samples with false positive (FP) variants. Error bars = 90% CI. Abbreviations: crc = colorectal cancer, nsclc = non-small cell lung cancer, cup = cancer of unknown primary (physician specified), gi- gastrointestinal, pns = paranasal sinus, cns = central nervous system, gist = gastrointestinal stromal tumor, FP = false positive, PPV = positive predictive value.

Results from analyzing historical tissue CGP data with the variant selection algorithm showed a median of 2 pathogenic alterations were identified per patient. When including all non-synonymous mutations within the coding region (pathogenic and VUS), there was a median of 3 alterations per patient. Adding synonymous and intronic alterations maximized the median number of monitorable alterations per patient to 6 (Fig 2A). Of all patients assessed, 90.0% had ≥2 monitorable alterations. The number of monitorable alterations per patient by disease type in our dataset are shown in Fig 2B. The inclusion of both coding and non-coding variants–regardless of known or likely pathogenicity–drastically increases the median number of alterations from tissue CGP per patient, as well as fraction of patients with at least two monitorable alterations, across all tumor types (S2A Fig). Thus, by including non-coding and non-pathogenic alterations the assay will be able to assess a large portion of patients previously diagnosed with cancer who have already been tested with tissue CGP.

The number of monitorable alterations per patient was consistent across key characteristics of tumor biology, such as local/met status and cancer stage (S3A–S3D Fig). Our ability to detect monitorable alterations did not differ among patients when stratified across predicted genetic ancestry (S3B Fig). The number of identified monitorable alterations per patient did increase with age and TMB status (S3C–S3E Fig).

Patient matched tissue and PBMCs were sequenced by tissue CGP and used to verify tumor origin of the monitorable alterations identified by the variant selection algorithm. A median of 11 monitorable alterations were selected per patient. Out of the 4588 variants selected 4535 were confirmed to be tumor-derived somatic alterations, resulting in a variant-level PPV of 98.8% [90% CI: 98.6%, 99.1%] (Fig 2C). There was no significant difference in PPV between pathogenic, VUS, or non-coding/synonymous alterations (S2B Fig). Variants detected in FFPE tissue that were not selected by the variant selection algorithm were not analyzed in this study. We predict those variants that were not selected are a mix of tumor-derived, CH, and germline (S1A Fig). For the other 53 variants, 17 were germline alterations and 36 were CH alterations. The inclusion of these non-tumor derived alterations could give rise to a false positive call in a ctDNA-negative patient. From the 477 patients that were tested, 5 patients had ≥2 non-tumor derived alterations selected for primer design resulting in a sample-level PPV of 99.0% [90% CI: 97.8%,99.6%] (Fig 2C). Thus, the variant selection algorithm has a high PPV for selecting tumor-derived somatic variants for monitoring, which is expected to translate into high specificity for ctDNA detection.

### Analytical validation

#### Plasma workflow specificity

**Fig 3:**
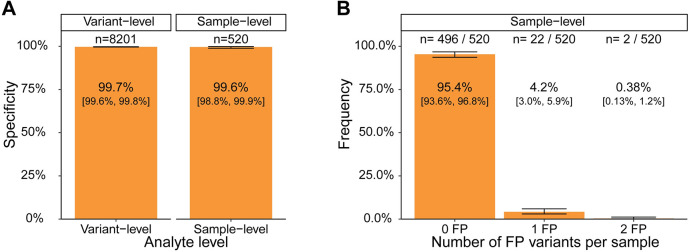
Specificity of plasma workflow. A) Specificity of plasma workflow at the variant and sample-level. B) Frequency of samples with false positive (FP) variants detected. Error bars = 90% CI.

To determine the false positive rate arising from technical sources (*i*.*e*., non-biological) in the plasma workflow, cfDNA from 133 unaffected donors were analyzed using primers designed from a separate set of cancer patient FFPE tumor samples. To generate the most conservative estimate of specificity, 16 monitorable alterations per sample were used. Pools of 16 alterations have the highest likelihood of having multiple false positives variants per pool. Variant-level specificity was determined by calculating the proportion of variants correctly called negative out of the 8201 tumor-associated variants assessed. 8175 variants were correctly called negative, giving a variant-level specificity of 99.7% [90% CI: 99.6%, 99.8%] (Fig 3A). Two out of the 520 unaffected donor samples each had 2 tumor-derived variants detected, leading to sample-level false positive calls (Fig 3B) and a sample-level specificity of 99.6% [90% CI: 98.8%, 99.9%] (Fig 3A).

#### Combined specificity estimate

The probability of a sample-level false positive occurring when one germline/CH variant is selected by the algorithm and a second false positive variant arises from technical sources was not captured in the specificity estimates above. When assessing the PPV of the variant selection algorithm, 41 patients (8.6%) had a single non-tumor-derived alteration designed (Fig 2D). The number of samples with ≥1 false positive variant from technical sources was calculated to be 4.6% [90% CI: 3.3%, 6.4%] (Fig 3B). Using these values and the sample-level specificity estimates above, we estimated the combined specificity of the variant selection and plasma workflow to be >96.0% (See Methods for calculation, point estimate = 98.2%). For validation purposes, this estimate used the most conservative bounds of each estimate to model specificity.

#### Analytical sensitivity

**Fig 4:**
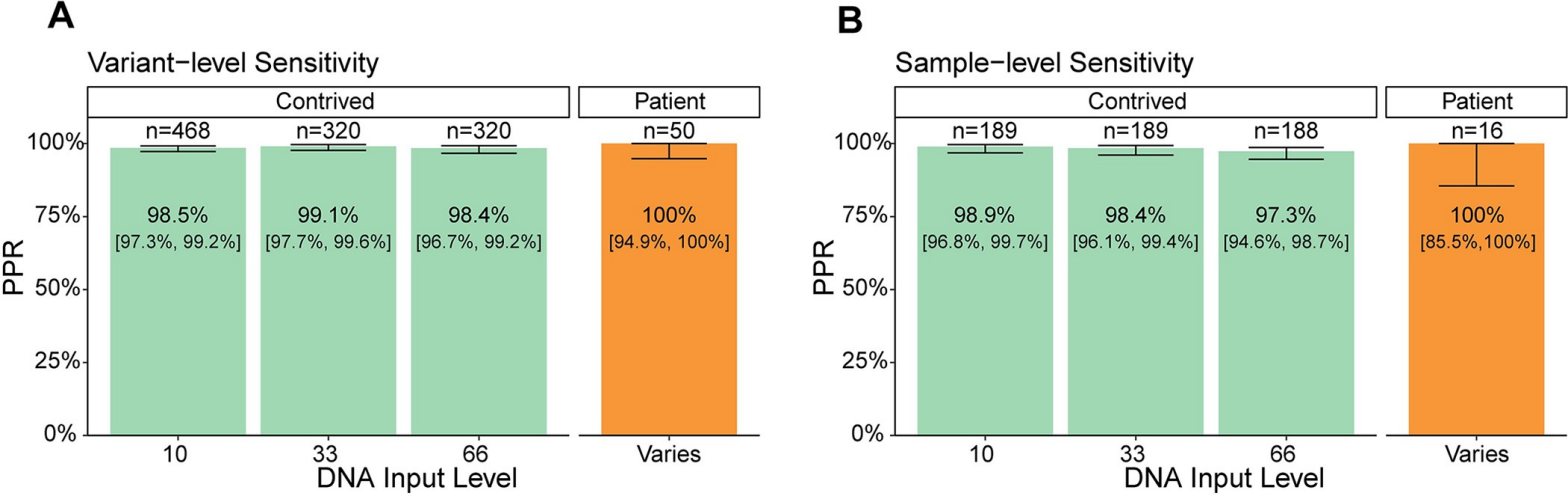
Sensitivity. A) PPR of plasma workflow at the variant-level for contrived (green) and patient (orange) samples. VAF range tested: ≥0.3% and < 0.5%. B) PPR of plasma workflow at the sample-level for contrived (green) and patient (orange) samples. MTM/mL range tested: ≥5 and <10 MTM/mL Error bars = 90% CI.

Analytical sensitivity of the assay was determined from contrived and patient samples that were diluted across a predefined VAF range that was representative of the majority of liquid biopsy samples with detected SNV or indel [33]. Variant-level sensitivity was calculated as the proportion of positive results (PPR) for variants with expected VAF ≥0.3% and <0.5%. The variant-level PPR for the 10, 33, and 66 ng DNA input levels were 98.5% [90% CI: 97.3%, 99.6%], 99.1% [90% CI: 97.7%,99.6%], and 98.44% [90% CI: 96.68%,99.24%] respectively (Fig 4A). To supplement the data from contrived samples, 16 patient samples were diluted into pooled unaffected donor cfDNA to target an expected diluted sample MTM/mL ≥5. From those samples, 50 variants were estimated to fall in the ≥0.3% and <0.5% range. All 50 variants were detected giving a PPR of 100% [90% CI: 94.9%, 100%] (Fig 4A) demonstrating the results for contrived samples are representative of the performance in patient samples.

Using *in silico* samples with 2 variants each, the sample-level PPR was computed separately for each DNA input level and all PPR were ≥97.3%. PPR for the 10, 33, and 66 ng DNA input levels were 98.9% [90% CI: 96.9%, 99.7%], 98.4% [90% CI: 96.1%, 99.4%], and 97.3% [90% CI: 94.6%,98.7%], respectively (Fig 4B). Thus, the assay has >94.6% (LB with 66ng input) sensitivity at the sample-level at the lower limit of detection, 5 MTM/mL. Of the 16 diluted patient samples tested, all were ctDNA positive, giving a sensitivity estimate of 100% [90% CI: 85.5%, 100%] (Fig 4B) demonstrating the results for contrived samples are representative of the performance in patient samples.

#### Accuracy and linearity

**Fig 5:**
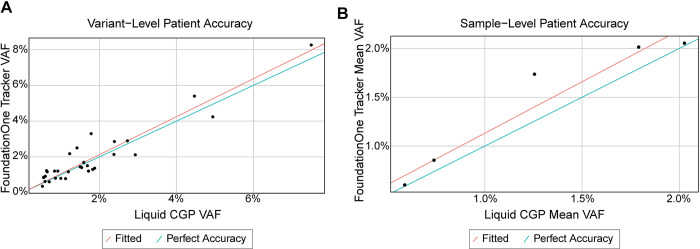
Accuracy using orthogonal method. A) Scatter plots of variant-level FoundationOne®Tracker (Y axis) vs liquid CGP (X axis) measured plasma VAF values for patient samples along with the fitted Passing-Bablok regression line (red) and a line indicating perfect fit (x = y, blue). B) Scatter plots of sample-level FoundationOne Tracker (Y axis) vs Liquid CGP (X axis) measured mean plasma VAF values for patient samples along with the fitted Passing-Bablok regression line (red) and a line indicating perfect fit (x = y, blue).

Liquid CGP was used as an orthogonal assay to assess agreement and possible systematic bias with FoundationOne®Tracker when measuring plasma VAF and mean VAF. Even though FoundationOne®Tracker clinically reports MTM/mL rather than individual VAFs or mean VAF, VAF was used for accuracy and linearity assessments because this measurement is available in an orthogonal validated assay and it is a key component of the derivation of MTM/mL for FoundationOne®Tracker. In the variant-level accuracy analysis for contrived samples, slope estimates for Passing-Bablok regression models fitted at each of the input levels falls within 0.046 of 1 (range: 1.038 to 1.046) and the respective intercept coefficient estimates all fall within -0.000874 of the predicted intercept of 0 (range: -0.000874 to -0.000451). Furthermore, R-squared values were ≥0.957, demonstrating the accuracy of the two methods at the variant-level (Table 2). Variant level linearity at each DNA input level was examined by fitting three different linear models. The coefficient estimates were examined for each different model fit and had a significant p-value (<0.05) for all 1^st^ order coefficients. Coefficients for 8/9 second and third order coefficients were >0.05, indicating a linear fit **(**S3 Table). 31 variants from patient samples (Fig 5A) showed similar agreement (R-squared = 0.975, slope = 1.063 and y-intercept = -0.0000373, Table 2) with the results for contrived samples.

**Table 2:** Slope and intercept regression coefficients from linearity study.

Assessment	DNA Input (ng)	# Observations	R-squared	Regression Coefficient	Point Estimate
**Variant-level Accuracy with contrived samples**	10ng	1924	0.965	Slope	1.046
				Intercept	-0.000874
	33ng	1922	0.960	Slope	1.038
				Intercept	-0.000533
	66ng	1922	0.957	Slope	1.038
				Intercept	-0.000451
**Sample-level Accuracy with contrived samples**	10ng	960	0.980	Slope	1.044
				Intercept	-0.000481
	33ng	951	0.976	Slope	1.032
				Intercept	-0.000219
	66ng	953	0.975	Slope	1.034
				Intercept	-0.000197
**Variant-level Accuracy with patient samples**	varies	31	0.975	Slope	1.063
				Intercept	-0.0000373
**Sample-level Accuracy with patient samples**	varies	5	0.982	Slope	1.054
				Intercept	0.0007802

Sample-level accuracy was assessed with 2-variant *in silico* samples simulated from the contrived sample accuracy data set to represent mean VAF range of 0.5–20%. In the sample-level accuracy analysis for contrived samples, slope estimates for Passing-Bablok regression models fitted at each of the input levels falls within 0.044 of 1 (range: 1.032 to 1.044) and the respective slope coefficient estimates all fall within -0.000874 of the predicted intercept of 0 (range: -000481 to -0.000197). Furthermore, R-squared values were ≥0.975, demonstrating accuracy at the sample-level (Table 2). The coefficient estimates were examined for each different model fit and had a significant p-value (<0.05) for all 1^st^ order coefficients. Coefficients for all second and third order coefficients were >0.05, indicating a linear fit **(**S3 Table). Results from 5 patient samples (Fig 5B) showed similar agreement (R-squared = 0.982, slope = 1.054 and y-intercept = 0.00078, Table 2) with the results for contrived samples.

#### Precision

**Fig 6:**
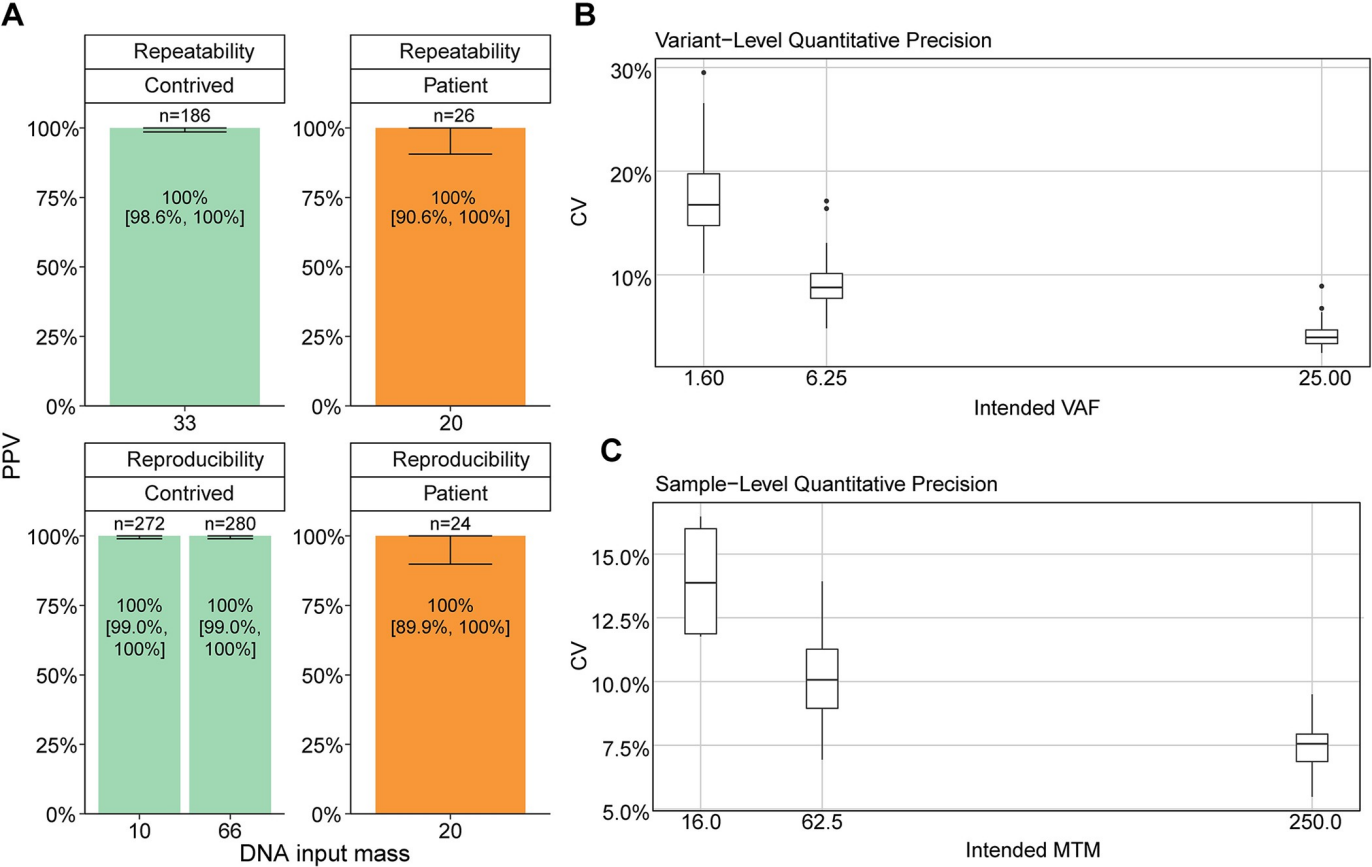
Precision. A) Variant-level repeatability and reproducibility for contrived (green) and patient (orange) samples separated by input mass. Error bars = 90% CI. C) Variant-level CV at multiple intended VAF and 90% bootstrap CI of overall mean CV. D) Sample-level CV at multiple intended MTM/mL and 90% bootstrap CI of overall mean CV.

Repeatability of qualitative variant detection was assessed with sample triplicates processed under the same conditions. The concordance proportion for contrived (n = 186) and patient samples (n = 26) was 100% [90% CI: 98.6%, 100%] (Fig 6A). Reproducibility of qualitative variant detection was assessed with sample triplicates processed under three sets of unique conditions. The concordance proportions for contrived samples at 10 ng input (n = 272) and 66 ng input (n = 280) were 100% [90% CI: 99.0%, 100%] and 100% [90% CI: 99.0%, 100%], respectively (Fig 6A). The mean CV was calculated as 10.23% [90% CI: 9.9%, 10.6%] at the variant-level and 10.5% [90% CI: 9.9%, 11.1%] at the sample-level (MTM/mL) (Fig 6B and 6C). Thus, the qualitative and quantitative results are highly reproducible and repeatable.

## Discussion

Detection and quantification of ctDNA in plasma is an emerging tool for detecting residual disease and informing treatment decisions. FoundationOne®Tracker is a tissue-informed ctDNA monitoring assay that combines the actionability of CGP with plasma-based mPCR for ctDNA detection and quantification. This assay provides a unique approach to monitoring by utilizing *in silico* germline and CH filtering. Based on our analysis of a wide range of tumor types, this approach is shown to be broadly applicable, including in a large portion of patients impacted by the most prevalent cancer types (Fig 2B). The plasma workflow of the assay has a specificity (i.e. FPs coming from non-biological sources) of >99.6% at the sample-level (Fig 3A) with a sensitivity of >97.3% at ≥5 MTM/mL in the conservative case where samples are tested with the minimum number of two monitorable alterations (Fig 4B). The assay also demonstrates high analytical accuracy when compared to liquid CGP (Fig 5) as well as high qualitative and quantitative precision (Fig 6). This assay clinically reports ctDNA levels as Mean tumor molecules per mL of plasma, which is a measurement that has been shown to have a high dynamic range, the ability to normalize for high background of cfDNA due to biological factors, and may be more predictive of patient outcomes [34]. However, other panel-based hybrid-capture assays measure ctDNA levels using VAF of tumor-derived variants or tumor fraction, which are both measurements of the ratio of ctDNA to background cfDNA. While a direct comparison between MTM/mL and Tumor fraction was not performed in this study, VAFs measured in FoundationOne®Tracker are highly correlated to Liquid CGP (Fig 5). Also MTM/mL as a measurement has been shown to be correlated to VAF previously [34].

One theoretical limitation of the assay is the efficiency of removing non-tumor-derived alterations computationally without utilizing sequencing of matched PBMC or normal tissue, thus potentially limiting specificity. Here we demonstrate the specificity of the variant selection algorithm to be 98.8% at the variant-level (Fig 2C). We calculated the combined variant selection and plasma workflow specificity to be >96.0% at the sample-level (calculation in methods section, PE = 98.2%). Both estimates of variant selection and plasma workflow specificity were done using the most conservative estimates for validation purposes. The specificity of the variant selection algorithm was calculated using a sample population with a median of 11 monitorable alterations per patient. Technical specificity was measured with 16 alterations per patient. Both are significantly higher than the median of 6 monitorable alterations per patient observed in the intended use population (Fig 2A). This illustrates that the assay has a high specificity while utilizing *in silico* germline filtering. However, to achieve this performance it is required to set the sample-level detection threshold at 2 alterations detected. We show that 8.6% of patients had 1 non-tumor-derived variant designed (Fig 2D) and 4.2% of samples had 1 false positive arise from technical sources (Fig 3B). Thus, the 2 alterations threshold optimizes specificity but could potentially lower sensitivity when compared to allowing the detection of a single alteration.

Selecting monitorable alterations from a 324-gene CGP panel could potentially identify a limited number of somatic alterations per sample as compared to WES or whole genome tissue sequencing. This limitation raises two concerns: 1) having fewer alterations could limit sensitivity of the assay and 2) that a limited number of patients would meet the requirement of having ≥2 monitorable alterations designed. Fewer monitorable alterations could limit sensitivity as the probability of detecting 2 would decrease. By designing the sensitivity study samples with 2 monitorable alterations, we sought to estimate lowest possible sensitivity of the assay and show it to be >97.3% in low ctDNA samples (5–10 MTM/mL). Sensitivity and precision of the assay will be less reliable at lower levels of ctDNA, however recent data suggests the assay is capable of detecting ctDNA down to the 0.4 MTM/mL level in metastatic colorectal cancer [2], and the performance of assay is sufficient to identify response to immunotherapy in patients with metastatic cancer [13,35] To address the possible limitation of evaluable patients, we show that 90.0% of patients with tissue CGP would have ≥2 monitorable alterations (Fig 2B). This was consistent among many of the most prevalent tumor types in our database, including NSCLC, CRC, and breast cancer. This is also consistent across metastatic status, ethnicity, stage, and TMB (S3 Fig). However, patients under 9 years of age had a median of <2 monitorable alterations (S3C Fig). Thus, the assay is likely applicable to a majority of non-pediatric cancers.

At the time of this writing no guidelines or protocols have been published for the analytical validation of tumor-informed personalized ctDNA monitoring and MRD assays, nor has any such assay been approved by a health authority with published safety and effectiveness data. Therefore, this validation was designed based on requirements from College of American Pathologists and New York State Department of Health for Lab developed tests and taking into account applicable recommendations for validation of cfDNA assays [36,37]. The studies described here are consistent with published recommendations for sample size, types of studies, and statistical methods, Future studies may be warranted if such MRD-specific guidelines become available.

The data described here support the analytical validity of CGP-informed ctDNA detection assays for late stage cancer patients. The results of these studies demonstrate the ability of the assay to quantify ctDNA accurately and with precision while demonstrating high sensitivity and specificity, thus providing a tool for clinicians to monitor patients.

## Supporting information

S1 Fig Variant selection algorithm. A) ROC curve demonstrating the True Positive Rate and False Positive Rates at differing somatic probability thresholds. Black dashed line indicates x = y. The vertical orange dashed line corresponds to a FPR of 0.01. B) Flowchart for the inclusion of short variant alterations in patient specific design.
(TIF)

S2 Fig Monitorable patients by tumor type. A) Fraction of patients with sufficient monitorable alterations from historical tissue CGP across tumor types. B) PPV from variant-level design (Fig 2C) separated by variant status. Abbreviations: crc = colorectal cancer, nsclc = non-small cell lung cancer, cup = cancer of unknown primary (physician specified), gi- gastrointestinal, pns = paranasal sinus, cns = central nervous system, gist = gastrointestinal stromal tumor, PPV = positive predictive value.
(TIF)

S3 Fig Number of monitorable alterations identified from historical tissue CGP across cancer and patient characteristics. A) Number of monitorable alterations across local versus metastatic sites. B) Number of monitorable alterations detected across patients from different predicted genetic ancestries. C) Number of monitorable alterations measured across age bins. D) Number of monitorable alterations detected from cancers stratified by physician-reported stage at the time of ordering the tissue CGP. E) Numbers of monitorable alterations in TMB high and TMB low patients, stratified by tumor type. Outlier points were removed from all boxplots for data interpretation. Dashed lines indicate 2 monitorable alterations on each plot.
(TIF)

S1 Table Mapping of disease ontology tumor types to disease groupings. (XLSX)

S2 Table Cell lines used to generate contrived samples. (XLSX)

S3 Table Linearity estimates from regression of accuracy study comparing FoundationOne®Tracker and liquid CGP. (XLSX)

S4 Table Supplementary datasets. (XLSX)

## References

[1] Vega DM, Nishimura KK, Zariffa N, Thompson JC, Hoering A, Cilento V, et al. Changes in Circulating Tumor DNA Reflect Clinical Benefit Across Multiple Studies of Patients With Non-Small-Cell Lung Cancer Treated With Immune Checkpoint Inhibitors. JCO Precis Oncol. 2022;6: e2100372. doi: 10.1200/PO.21.00372 35952319 PMC9384957

[2] Lonardi S, Nimeiri H, Xu C, Zollinger DR, Madison RW, Fine AD, et al. Comprehensive Genomic Profiling (CGP)-Informed Personalized Molecular Residual Disease (MRD) Detection: An Exploratory Analysis from the PREDATOR Study of Metastatic Colorectal Cancer (mCRC) Patients Undergoing Surgical Resection. Int J Mol Sci. 2022;23. doi: 10.3390/ijms231911529 36232827 PMC9569771

[3] Sanz-Garcia E, Zhao E, Bratman S V, Siu LL. Monitoring and adapting cancer treatment using circulating tumor DNA kinetics: Current research, opportunities, and challenges. Sci Adv. 2022;8: eabi8618. doi: 10.1126/sciadv.abi8618 35080978 PMC8791609

[4] Cescon DW, Bratman S V, Chan SM, Siu LL. Circulating tumor DNA and liquid biopsy in oncology. Nat Cancer. 2020;1: 276–290. doi: 10.1038/s43018-020-0043-5 35122035

[5] Evison M, Barrett E, Cheng A, Mulla A, Walls G, Johnston D, et al. Predicting the Risk of Disease Recurrence and Death Following Curative-intent Radiotherapy for Non-small Cell Lung Cancer: The Development and Validation of Two Scoring Systems From a Large Multicentre UK Cohort. Clin Oncol (R Coll Radiol). 2021;33: 145–154. doi: 10.1016/j.clon.2020.09.001 32978027

[6] Woodard GA, Kratz JR, Haro G, Gubens MA, Blakely CM, Jones KD, et al. Molecular Risk Stratification is Independent of EGFR Mutation Status in Identifying Early-Stage Non-Squamous Non-Small Cell Lung Cancer Patients at Risk for Recurrence and Likely to Benefit From Adjuvant Chemotherapy. Clin Lung Cancer. 2021;22: 587–595. doi: 10.1016/j.cllc.2021.08.008 34544620

[7] Tie J, Cohen JD, Wang Y, Christie M, Simons K, Lee M, et al. Circulating Tumor DNA Analyses as Markers of Recurrence Risk and Benefit of Adjuvant Therapy for Stage III Colon Cancer. JAMA Oncol. 2019;5: 1710–1717. doi: 10.1001/jamaoncol.2019.3616 31621801 PMC6802034

[8] Chaudhuri AA, Chabon JJ, Lovejoy AF, Newman AM, Stehr H, Azad TD, et al. Early Detection of Molecular Residual Disease in Localized Lung Cancer by Circulating Tumor DNA Profiling. Cancer Discov. 2017;7: 1394–1403. doi: 10.1158/2159-8290.CD-17-0716 28899864 PMC5895851

[9] Caswell-Jin JL, Callahan A, Purington N, Han SS, Itakura H, John EM, et al. Treatment and Monitoring Variability in US Metastatic Breast Cancer Care. JCO Clin Cancer Inform. 2021;5: 600–614. doi: 10.1200/CCI.21.00031 34043432 PMC8462601

[10] Eisenhauer EA, Therasse P, Bogaerts J, Schwartz LH, Sargent D, Ford R, et al. New response evaluation criteria in solid tumours: revised RECIST guideline (version 1.1). Eur J Cancer. 2009;45: 228–47. doi: 10.1016/j.ejca.2008.10.026 19097774

[11] Garlan F, Laurent-Puig P, Sefrioui D, Siauve N, Didelot A, Sarafan-Vasseur N, et al. Early Evaluation of Circulating Tumor DNA as Marker of Therapeutic Efficacy in Metastatic Colorectal Cancer Patients (PLACOL Study). Clin Cancer Res. 2017;23: 5416–5425. doi: 10.1158/1078-0432.CCR-16-3155 28576867

[12] Schwartz LH, Litière S, de Vries E, Ford R, Gwyther S, Mandrekar S, et al. RECIST 1.1-Update and clarification: From the RECIST committee. Eur J Cancer. 2016;62: 132–7. doi: 10.1016/j.ejca.2016.03.081 27189322 PMC5737828

[13] Kansara M, Bhardwaj N, Thavaneswaran S, Xu C, Lee JK, Chang L-B, et al. Early circulating tumor DNA dynamics as a pan-tumor biomarker for long-term clinical outcome in patients treated with durvalumab and tremelimumab. Mol Oncol. 2023;17: 298–311. doi: 10.1002/1878-0261.13349 36426653 PMC9892824

[14] Zhang Q, Luo J, Wu S, Si H, Gao C, Xu W, et al. Prognostic and Predictive Impact of Circulating Tumor DNA in Patients with Advanced Cancers Treated with Immune Checkpoint Blockade. Cancer Discov. 2020;10: 1842–1853. doi: 10.1158/2159-8290.CD-20-0047 32816849 PMC8358981

[15] Reinert T, Henriksen TV, Christensen E, Sharma S, Salari R, Sethi H, et al. Analysis of Plasma Cell-Free DNA by Ultradeep Sequencing in Patients With Stages I to III Colorectal Cancer. JAMA Oncol. 2019;5: 1124–1131. doi: 10.1001/jamaoncol.2019.0528 31070691 PMC6512280

[16] Ko TK, Lee E, Ng CC-Y, Yang VS, Farid M, Teh BT, et al. Circulating Tumor DNA Mutations in Progressive Gastrointestinal Stromal Tumors Identify Biomarkers of Treatment Resistance and Uncover Potential Therapeutic Strategies. Front Oncol. 2022;12: 840843. doi: 10.3389/fonc.2022.840843 35273917 PMC8904145

[17] Gale D, Heider K, Ruiz-Valdepenas A, Hackinger S, Perry M, Marsico G, et al. Residual ctDNA after treatment predicts early relapse in patients with early-stage non-small cell lung cancer. Ann Oncol. 2022;33: 500–510. doi: 10.1016/j.annonc.2022.02.007 35306155 PMC9067454

[18] Davis AA, Iams WT, Chan D, Oh MS, Lentz RW, Peterman N, et al. Early Assessment of Molecular Progression and Response by Whole-genome Circulating Tumor DNA in Advanced Solid Tumors. Mol Cancer Ther. 2020;19: 1486–1496. doi: 10.1158/1535-7163.MCT-19-1060 32371589 PMC8142024

[19] Klein EA, Richards D, Cohn A, Tummala M, Lapham R, Cosgrove D, et al. Clinical validation of a targeted methylation-based multi-cancer early detection test using an independent validation set. Annals of Oncology. 2021;32: 1167–1177. doi: 10.1016/j.annonc.2021.05.806 34176681

[20] Deveson IW, Gong B, Lai K, LoCoco JS, Richmond TA, Schageman J, et al. Evaluating the analytical validity of circulating tumor DNA sequencing assays for precision oncology. Nat Biotechnol. 2021;39: 1115–1128. doi: 10.1038/s41587-021-00857-z 33846644 PMC8434938

[21] Newman AM, Bratman S V., To J, Wynne JF, Eclov NCW, Modlin LA, et al. An ultrasensitive method for quantitating circulating tumor DNA with broad patient coverage. Nat Med. 2014;20: 548–554. doi: 10.1038/nm.3519 24705333 PMC4016134

[22] Moding EJ, Nabet BY, Alizadeh AA, Diehn M. Detecting Liquid Remnants of Solid Tumors: Circulating Tumor DNA Minimal Residual Disease. Cancer Discov. 2021;11: 2968–2986. doi: 10.1158/2159-8290.CD-21-0634 34785539 PMC8976700

[23] Lanman RB, Mortimer SA, Zill OA, Sebisanovic D, Lopez R, Blau S, et al. Analytical and Clinical Validation of a Digital Sequencing Panel for Quantitative, Highly Accurate Evaluation of Cell-Free Circulating Tumor DNA. PLoS One. 2015;10: e0140712. doi: 10.1371/journal.pone.0140712 26474073 PMC4608804

[24] Woodhouse R, Li M, Hughes J, Delfosse D, Skoletsky J, Ma P, et al. Clinical and analytical validation of foundation one liquid CDx, a novel 324-Gene cfDNA-based comprehensive genomic profiling assay for cancers of solid tumor origin. PLoS One. 2020;15. doi: 10.1371/journal.pone.0237802 32976510 PMC7518588

[25] Milbury CA, Creeden J, Yip WK, Smith DL, Pattani V, Maxwell K, et al. Clinical and analytical validation of FoundationOne®CDx, a comprehensive genomic profiling assay for solid tumors. PLoS One. 2022;17. doi: 10.1371/journal.pone.0264138 35294956 PMC8926248

[26] Frampton GM, Fichtenholtz A, Otto GA, Wang K, Downing SR, He J, et al. Development and validation of a clinical cancer genomic profiling test based on massively parallel DNA sequencing. Nat Biotechnol. 2013;31: 1023–1031. doi: 10.1038/nbt.2696 24142049 PMC5710001

[27] Loupakis F, Sharma S, Derouazi M, Murgioni S, Biason P, Rizzato MD, et al. Detection of Molecular Residual Disease Using Personalized Circulating Tumor DNA Assay in Patients With Colorectal Cancer Undergoing Resection of Metastases. JCO Precis Oncol. 2021;5. doi: 10.1200/PO.21.00101 34327297 PMC8315303

[28] Weinberg BA, Winslow ER, Bayasi M, Krainock MR, Olshan PM, Billings PR, et al. Early Detection of Circulating Tumor DNA Postoperatively Enables Discovery of Resectable Metastatic Disease in a Patient with Colon Cancer. Case Rep Oncol. 2021;14: 1748–1753. doi: 10.1159/000520743 35082635 PMC8739946

[29] Chalmers ZR, Connelly CF, Fabrizio D, Gay L, Ali SM, Ennis R, et al. Analysis of 100,000 human cancer genomes reveals the landscape of tumor mutational burden. Genome Med. 2017;9: 34. doi: 10.1186/s13073-017-0424-2 28420421 PMC5395719

[30] Connelly C, Carrot-Zhang J, Stephens P, Frampton G. Abstract 1227: Somatic genome alterations in cancer as compared to inferred patient ancestry. Cancer Res. 2018;78: 1227. doi: 10.1158/1538-7445.AM2018-1227

[31] Abbosh C, Birkbak NJ, Wilson GA, Jamal-Hanjani M, Constantin T, Salari R, et al. Phylogenetic ctDNA analysis depicts early-stage lung cancer evolution. Nature. 2017;545: 446–451. doi: 10.1038/nature22364 28445469 PMC5812436

[32] Powles T, Assaf ZJ, Davarpanah N, Banchereau R, Szabados BE, Yuen KC, et al. ctDNA guiding adjuvant immunotherapy in urothelial carcinoma. Nature. 2021;595: 432–437. doi: 10.1038/s41586-021-03642-9 34135506

[33] Husain H, Pavlick DC, Fendler BJ, Madison RW, Decker B, Gjoerup O, et al. Tumor Fraction Correlates With Detection of Actionable Variants Across > 23,000 Circulating Tumor DNA Samples. JCO Precis Oncol. 2022;6: e2200261. doi: 10.1200/PO.22.00261 36265119 PMC9616642

[34] Kalashnikova E, Aushev VN, Malashevich AK, Tin A, Krinshpun S, Salari R, et al. Correlation between variant allele frequency and mean tumor molecules with tumor burden in patients with solid tumors. Mol Oncol. 2023. 10.1002/1878-0261.13557. PMC11547219 38037739

[35] Pellini B, Madison RW, Childress MA, Miller ST, Gjoerup O, Cheng J, et al. Circulating Tumor DNA Monitoring on Chemo-immunotherapy for Risk Stratification in Advanced Non-Small Cell Lung Cancer. Clin Cancer Res. 2023; 29(22):4596–4605. doi: 10.1158/1078-0432.CCR-23-1578 37702716 PMC10643998

[36] Lockwood CM, Borsu L, Cankovic M, Earle JSL, Gocke CD, Hameed M, et al. Recommendations for Cell-Free DNA Assay Validations: A Joint Consensus Recommendation of the Association for Molecular Pathology and College of American Pathologists. J Mol Diagn. 2023 Dec;25(12):876–897. doi: 10.1016/j.jmoldx.2023.09.004 37806433

[37] Godsey JH, Silvestro A, Barrett JC, Bramlett K, Chudova D, Deras I, et al. Generic Protocols for the Analytical Validation of Next-Generation Sequencing-Based ctDNA Assays: A Joint Consensus Recommendation of the BloodPAC’s Analytical Variables Working Group. Clin Chem. 2020 Sep 1;66(9):1156–1166. doi: 10.1093/clinchem/hvaa164 32870995 PMC7462123

