# Supplementary figures and images for: Analytical validation of a novel comprehensive genomic profiling informed circulating tumor DNA monitoring assay for solid tumors
Source: PLoS One. 2024 May 16;19(5):e0302129. doi: 10.1371/journal.pone.0302129 (PMC11098318; doi:10.1371/journal.pone.0302129)

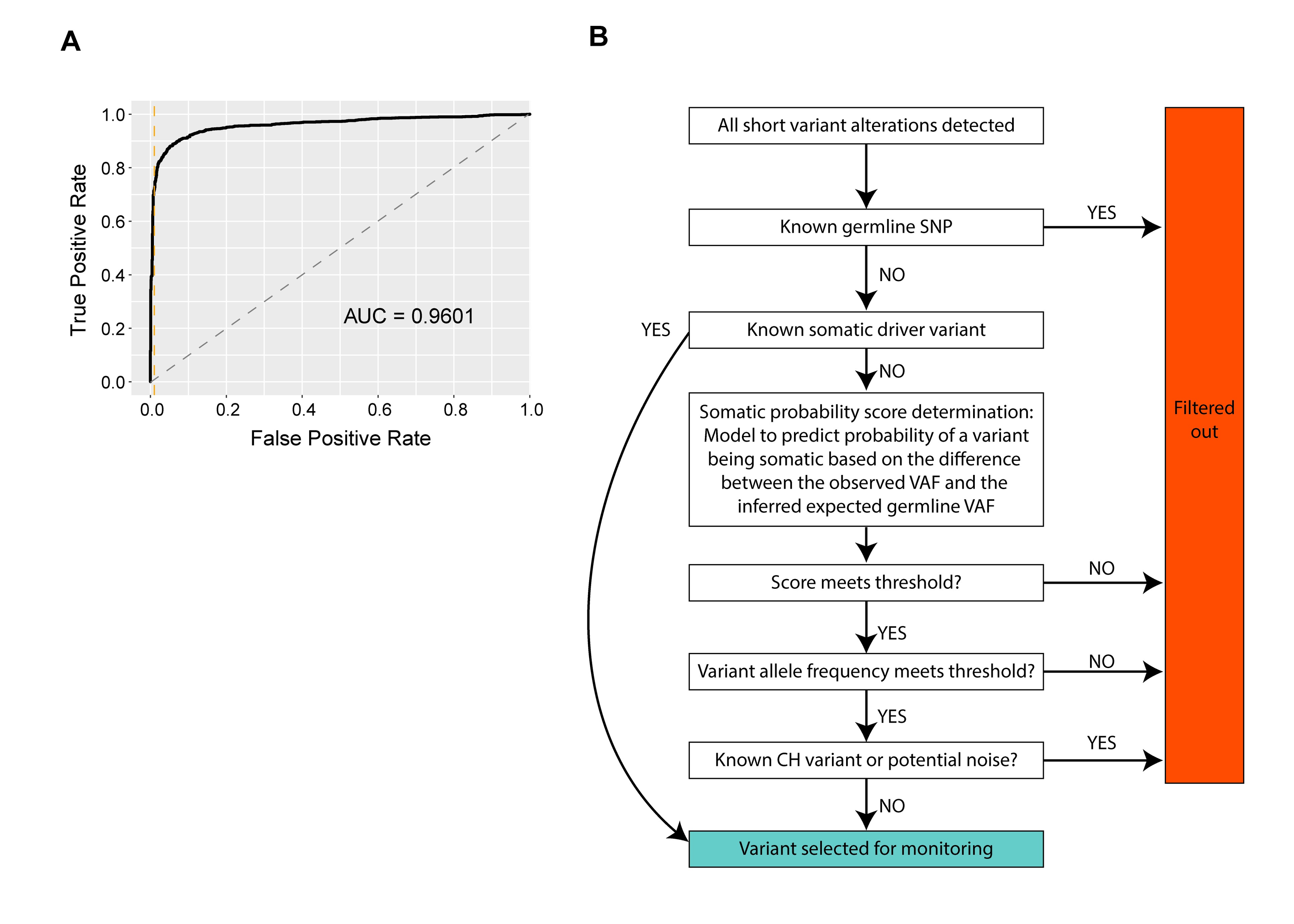

Supplement: S1 Fig — A) ROC curve demonstrating the True Positive Rate and False Positive Rates at differing somatic probability thresholds. Black dashed line indicates x = y. The vertical orange dashed line corresponds to a FPR of 0.01. B) Flowchart for the inclusion of short variant alterations in patient specific design. (TIF) [file pone.0302129.s001.tif]

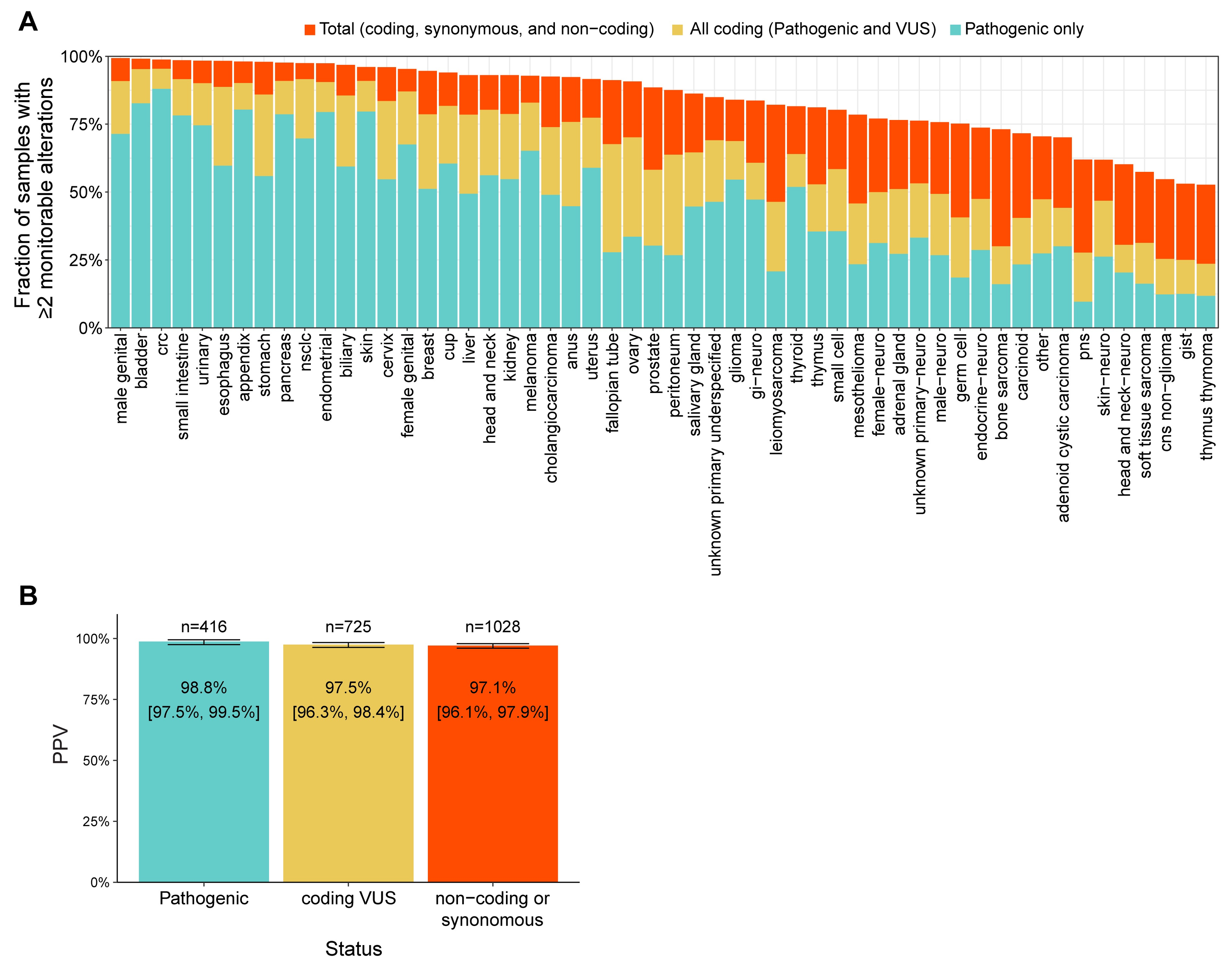

Supplement: S2 Fig — A) Fraction of patients with sufficient monitorable alterations from historical tissue CGP across tumor types. B) PPV from variant-level design (Fig 2C) separated by variant status. Abbreviations: crc = colorectal cancer, nsclc = non-small cell lung cancer, cup = cancer of unknown primary (physician specified), gi- gastrointestinal, pns = paranasal sinus, cns = central nervous system, gist = gastrointestinal stromal tumor, PPV = positive predictive value. (TIF) [file pone.0302129.s002.tif]

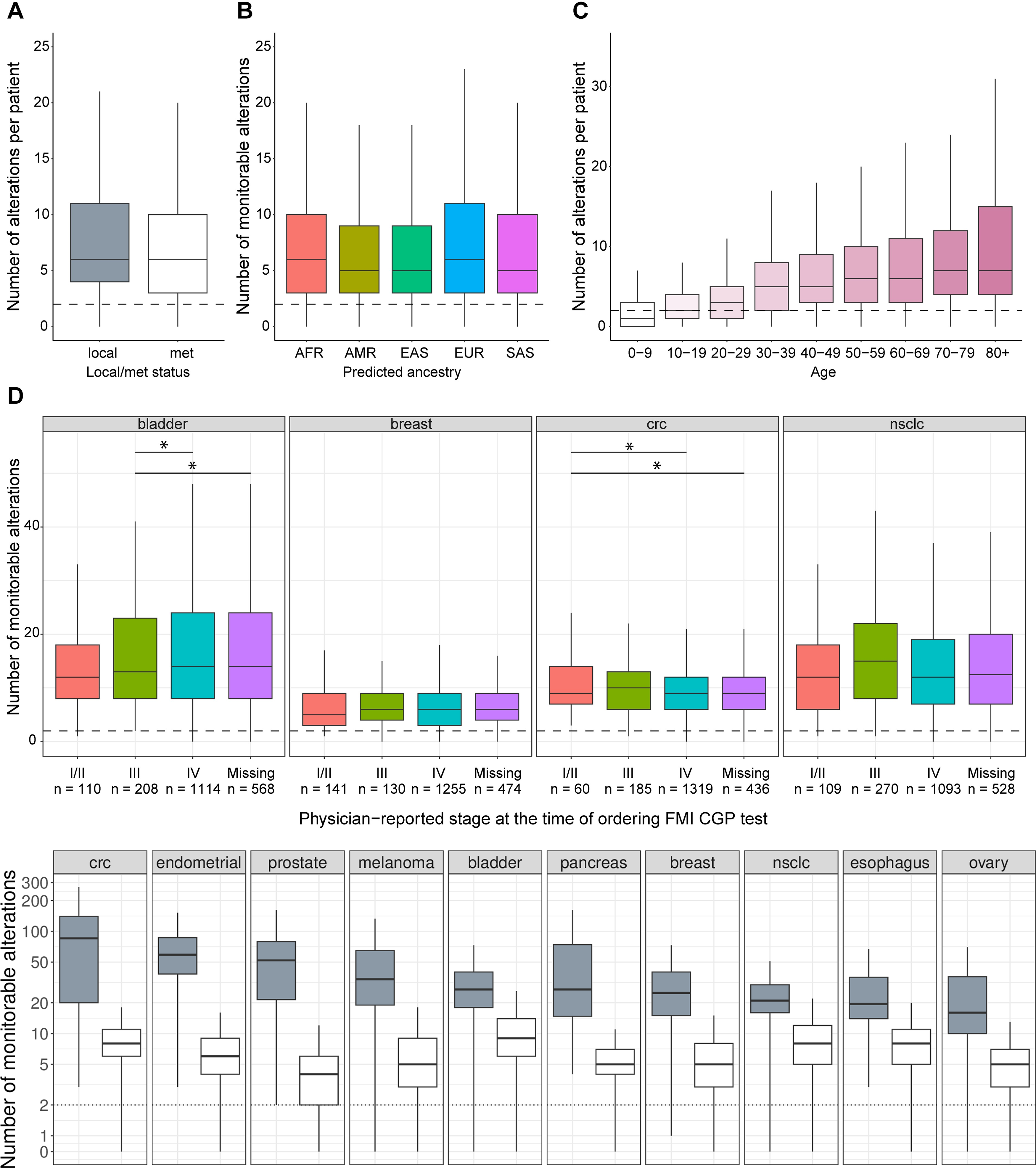

Supplement: S3 Fig — A) Number of monitorable alterations across local versus metastatic sites. B) Number of monitorable alterations detected across patients from different predicted genetic ancestries. C) Number of monitorable alterations measured across age bins. D) Number of monitorable alterations detected from cancers stratified by physician-reported stage at the time of ordering the tissue CGP. E) Numbers of monitorable alterations in TMB high and TMB low patients, stratified by tumor type. Outlier points were removed from all boxplots for data interpretation. Dashed lines indicate 2 monitorable alterations on each plot. (TIF) [file pone.0302129.s003.tif]
